# Institutional hybridity and policy-motivated reasoning structure public evaluations of the Supreme Court

**DOI:** 10.1371/journal.pone.0294525

**Published:** 2023-11-22

**Authors:** Shana Kushner Gadarian, Logan Strother

**Affiliations:** 1 Department of Political Science, Syracuse University, Syracuse, New York, United States of America; 2 Department of Political Science, Purdue University, West Lafayette, Indiana, United States of America; University of Foggia: Universita degli Studi di Foggia, ITALY

## Abstract

How does the public assess the Supreme Court and its work? Using data from three surveys conducted over a span of ten years, we show that individuals’ policy preferences drive evaluations of the Court and its willingness to reform the Court. We find strong evidence that the Court’s hybrid legal-political nature enables a unique form of policy-motivated reasoning: respondents who agree with Court outputs view the Court and its work as more “legal” in nature, while those who disagree view both as more “political.” Our findings stand in contrast to longstanding views in the literature that the public views the Court as a fundamentally different sort of institution that stands largely separate from politics. The fact that policy attitudes powerfully inform the public’s assessment of the Court has crucial implications for the ongoing debates over Supreme Court power.

## Introduction

Polarization in American politics has substantially increased the prominence of the Supreme Court of the United States (SCOTUS) as a policymaking institution [1]. At the same time as the Court’s centrality in politics has grown, it has also become considerably more conservative—much more so than the general public [2]. This raises a crucial question: does the mismatch between policy outputs of the Court and the policy preferences of the American people threaten to undermine public support for the Court? This question is important because the Supreme Court lacks the ability to enforce a large fraction of its decisions, and as such depends on public support for its efficacy as a political institution.

A growing body of work suggests that the answer to this question is “yes”–people evaluate the Court, in important part, based on agreement with policy outcomes from the Court’s decisions [3–5]. To date, we lack any clear account of why and how policy preferences inform institutional assessments. We show that a form of policy-motivated reasoning, enabled by the Court’s unusual hybrid structure, explains the relationship between the public’s policy attitudes and assessments of the Court as an institution. We contribute to the literature on the Supreme Court by tracing out the pathway that policy attitudes take to inform public sentiment about the Court and how design of the Court itself facilitates this type of policy reasoning. Our argument brings institutional context more fully into the literature on motivated reasoning. Motivated reasoning can happen through multiple pathways–undermining sources of information, refusing to update facts, attributing facts different attributes of responsibility. Here, we demonstrate that with a non-elected institution, there is another route for this reasoning, through denigrating the outcome as “political.”

Unlike the representative branches of government in the American political system, that are clearly designed to be political and responsive to the public, the Supreme Court is a unique institution that is simultaneously legal and political in nature [6]. The public recognizes this reality [5–7], although they think the Court *should* be apolitical [8]. The justices themselves care about how the Court is perceived by the public, routinely emphasizing the fundamentally fair, legal, nature of their work and going to pains to distinguish it from “politics” in their public speeches and interviews [9].

We argue that the reality of the Court’s institutional hybridity, along with the widespread preference that the Court behaves as a legal rather than political institution, combine to enable a type of motivated reasoning that shapes how the public evaluates the Court. In this model, the public uses its preferred policy outcomes as a guide to assess 1) individual Court decisions and in turn, 2) the Court itself. Specifically, people can believe that Court decisions they agree with are based on law and fair legal processes, and simultaneously believe that outcomes they disagree with are due to “politics.” This allows the public to maintain an overall favorable view of the Court even when it makes individual decisions they disagree with so long as everyone feels like they are “winning” before the Court at least some of the time. Yet, if the Court continually makes policy decisions out of line with public sentiment, we expect that the accumulation of policy losses will lead the public to perceive the Court as acting more like “politicians in robes,” in the words of retired Justice Stephen Breyer, and to downgrade approval of Court as a whole [10,11].

We are far from the first to argue that policy preferences inform attitudes toward the Court. Prior work has argued that there are important partisan and ideological components to public assessments of Supreme Court decisions [3,4], although the prevailing view in the literature is that partisan and ideological preferences only weakly inform views of the Court [12,13]. There is merit to both arguments. In most prior studies, scholars have assumed (or asserted) respondents’ policy views from the ideology. That is, if a Supreme Court decision is conservative and a respondent identifies as conservative, scholars assume that the respondent agrees with the decision. Partisanship and ideology correlate only weakly with policy preferences among members of the mass public, however [14]. As such, prior studies have imperfectly captured the effects of policy preferences on public assessments of Court decisions and judicial institutions [5]. As a result, we argue that these prior works may have substantially underestimated the extent to which policy preferences shape views of the Court as an institution, job approval, and trust in its decision-making.

In this paper, we draw on evidence from three national surveys, including one with an embedded experiment, to show that this policy-based motivated reasoning is a key driver of public assessments of the Supreme Court. Consistent with expectations, we find that when people agree with the Court’s decisions, they are more likely to view that decision as being based in “the law,” and when they disagree they are more likely to view it as “political.” Additionally, we find that people who disagree with decisions show less support for the Court and are more likely to support institutional reforms. In an original survey experiment, we show strong evidence for our policy motivated reasoning account of the linkages between policy disagreement and attitudes toward the Supreme Court and its work.

These findings have crucial implications for contemporary politics. If a large fraction of the public sees the Court’s decision as increasingly based on political rather than legal grounds, we expect that they will support efforts to curb the Court’s power or reform it to address the (perceived) problem.

In the opening statement of his confirmation hearing, John Roberts drew an analogy between judging and umpiring: “Judges are like umpires. Umpires don’t make the rules; they apply them. The role of an umpire and a judge is critical. They make sure everybody plays by the rules. But it is a limited role. Nobody ever went to a ball game to see the umpire… I will remember that it’s my job to call balls and strikes and not to pitch or bat” [15]. The umpire analogy, to work as Roberts wants it to, requires us to assume that “the rules” to be applied are objective and uncontested. But this is not the world that real judges nor umpires inhabit.

Consider what is typically the most visible act of umpiring: calling balls and strikes. The strike zone is, in theory, objective: “The official strike zone is the area over home plate from the midpoint between a batter’s shoulders and the top of the uniform pants–when the batter is in his stance and prepared to swing at a pitched ball–and a point just below the kneecap” [16]. The theoretical strike zone is clear and easy to understand, but some pitches, especially near the edges of the zone, can be hard to discern even for professional umpires. Umpires sometimes miss what appear to be easy calls—calling a strike on a pitch several inches low and away, for example. Sometimes, umpires will call entire games where the functional strike zone bears only passing resemblance to the one described in the rules.

Judges, in a similar way, must interpret and apply rules—though often the rules they are tasked with enforcing are sometimes much less clear than the MLB strike zone [17]. And like umpires, they are often presented with very difficult edge cases. Sometimes judges get it wrong in what appear to be easy cases or appear to apply rules unevenly across cases.

That both judges and umpires sometimes make bad calls points to another useful similarity unintentionally evoked in Roberts’ metaphor. “Nobody ever went to a ball game to see the umpire,” Roberts says. But this does not mean people in the ballpark are not interested in what the umpires are doing or how well they are doing it. Fans can see the strike zone too, although the view from the stands is not as clear as the one the umpire enjoys. Even so, when the home team is batting, the fans are confident that those edge pitches are balls; when the visitors bat, those same pitches appear to the fans to be strikes. What fans want is for their team to win. In their eyes, a “good” call is one that benefits their team [18].

Turning back to judges, the analog to the fan is the citizen with policy preferences. Observers on the left and right prefer procedural fairness, and both expect judges to apply the rules as they see them [19]. Much like baseball fans, Americans want the judges—and especially that paragon of law, the Supreme Court—to make *good* decisions. Good decisions, once again, are often those that comport with the policy preferences of a given observer.

Motivated reasoning affects this phenomenon in baseball fans and Court-watchers alike. In forming attitudes, people are motivated to both be accurate and consistent with prior beliefs and preferences [20,21]. These motivations affect information processing, particularly when views are deeply held. When people are motivated to be accurate, they will spend more effort in cognition, evaluate information more carefully and process it more deeply [21]. When people are more motivated by directional goals–that is, they want to arrive at an outcome they like, they do not simply accept information that would cause them to change their beliefs. Instead, they can counterargue, criticize the source, or selectively remember or encode countervailing information, which can provide justifications for their beliefs [22–25]. Even with similar or identical facts, partisans can come to different interpretations [26] or misattribute responsibility for facts to align beliefs with preferences [27]. Our argument brings institutional context more fully into the literature on motivated reasoning.

We argue that people process that information in a way that both accommodates the desire to have decisions that are in line with their preferred policy (e.g., nationwide legalized abortion) as well as maintains their view of the Court as a neutral arbiter of law. Support for the Supreme Court is higher and generally more robust than support for other institutions of government [28]. Previous literature argues that this deep well of support for the Court is challenging to undermine because both the Court itself and media coverage of Court decisions reinforce the view of the Court as broadly apolitical [8]. Even despite this deep diffuse support, public support for the Court is sensitive to the outcome of cases, particularly when the cases garner a high degree of media coverage and media framing highlights the ideological or non-legal aspects of the decision [3,4]. In the case of opinions about the Supreme Court, people take advantage of the fact of the Court’s hybrid legal-political nature to engage in a unique type of motivated reasoning based on what policy outcomes they want and attribute Court outcomes to either law or politics [29]. So, when faced with new Court decisions, we hypothesize that *the public deems decisions they agree with (i*.*e*., *“good decisions”) as being legal opinions and those that they disagree with (i*.*e*., *“bad decisions”) as coming from politics*. We expect, in turn, that *those who see the Court and its decisions as more “political” (less “legal”) will be more supportive of judicial reform and court curbing measures*.

We also hypothesize that *the effects of policy attitudes will matter most when these issues are salient in media coverage of the Court’s docket*. While there is less media coverage of the Supreme Court than other branches [30], mass media coverage of the Supreme Court tends to focus on cases that are both politically and legally important [31]. In addition, features of cases also drivemedia coverage–cases that overturn precedents, reverse lower courts, and have judicial dissensus are more likely to be covered [32], and issue areas like civil rights garner more attention [33]. One implication of the drive toward covering “newsworthy cases” is that even potentially prominent policy areas discussed in previous Court terms are unlikely to be covered by the media unless they are related to a current case. That is, when policy preferences are top of mind, they are more likely to shape evaluations of Court decisions and the Court itself.

This mode of evaluating the Court has substantial real-world implications. As the Court becomes more conservative (or liberal) relative to the public, people with liberal (conservative) policy views will receive fewer policy wins from the Court over time [2,4]. As losses pile up for a fan’s favorite team and wins for their opponents stack up, the fan will call for the umpires to be fired. This brings us to our final hypothesis: we expect that *people who disagree with decisions will view the Court itself as more “political” (and less “legal”) in nature than those who agree with its decisions*.

## Results

We draw on three data sources to test our model of public assessments of the Supreme Court, each using different operationalizations of attitudes toward the Court. The data are from the American National Election Studies (ANES), the UMass-Amherst Poll, and an online non-probability sample provided by SSI/ResearchNow. All of the statistical tests we present are two-tailed. We find strong support for our policy motivated reasoning account across all 3 studies.

### Study 1: The Affordable Care Act and attitudes toward SCOTUS in 2012

In Study 1, we trace out the impact of policy attitudes on evaluations of the Court as a whole. We use data from the 2012 American National Election Studies, a nationally representative sample of close to 5,000 Americans, to assess the influence of the Supreme Court’s highest-profile decision that year on respondents’ feelings toward the Court, measured as warmth toward the institution. We expect people to evaluate the Court based on how well decisions comport with their policy preferences on the salient policy issues implicated by the Court’s decisions. In June of 2012, the Court issued its decision in *NFIB v*. *Sebelius* (2012, 567 US ___), in which a 5–4 majority upheld the Affordable Care Act (ACA, or “Obamacare”) as constitutional. This decision was celebrated by those who favor a role for government in healthcare, and reviled by others, who prefer a wholly private market. The survey was in the field from September of 2012 until January of 2013, allowing us to assess possible relationships between policy attitudes and Court decisions including *NFIB v*. *Sebelius*.

We expect that those respondents who support the ACA would feel more warmly toward the Court than those who opposed the ACA. We model the impact of ACA attitudes on assessments of the Court, controlling for partisan identification, political ideology, race, gender, and education using OLS regression. Unadjusted models are presented in the supplement. We capture attitudes toward the Court in two ways–a feeling thermometer toward the Court and an index that indicates a willingness to curb the Court’s power.

**Fig 1:**
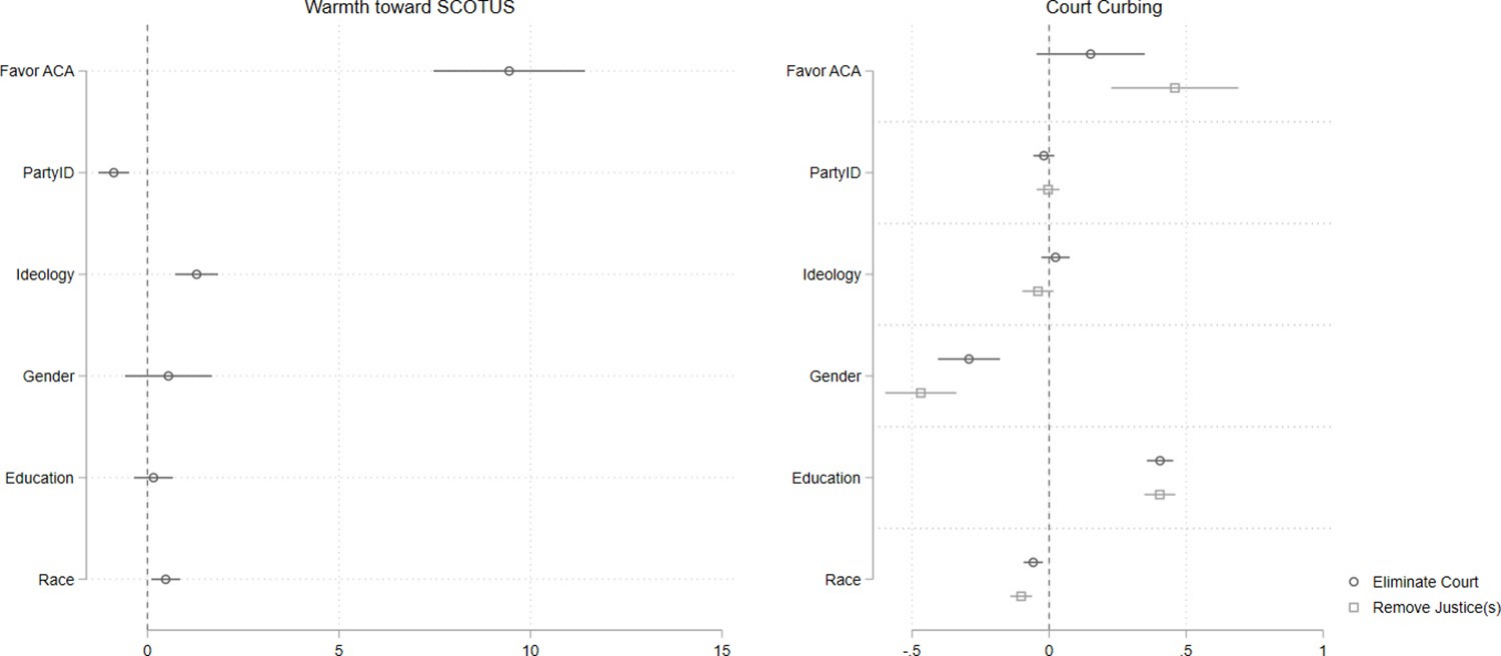
Favorability of affordable care act predicts attitudes toward SCOTUS in 2012. Point estimates with 95% confidence intervals from multivariate OLS models. Estimates in panel at left are based on *n =* 4,802 respondents. Estimates in panel at right are based on *n =* 4,757 (“eliminate court”) and *n =* 4,722 (“remove justice(s)).

Fig 1 depicts the findings from these separate OLS models graphically. The full models are presented in the supplement. First, in the panel at left, we show that warmth toward the Court is highly correlated with individuals’ policy preferences regarding the ACA, net of controls. Those who strongly favor the ACA report being roughly 10 points warmer toward SCOTUS compared to those who are opposed to the ACA. Next, we move to alternative dependent variables that capture support for Supreme Court reform. The first of these asks respondents whether the Supreme Court should be eliminated; the second asks whether it should be possible to remove Supreme Court justices (both variables are coded such that higher values correspond to more “pro-Court” attitudes, that is *opposition* to reform). The panel at right in Fig 1 shows that people who held favorable opinions toward the ACA were significantly more opposed to removing justices than those who opposed the Court’s decision. Note that in both analyses it is specific policy preferences, net of partisanship and ideology, that explain the outcomes of interest. This is especially notable given the relatively strong and clear partisan dimension of the politics surrounding the Affordable Care Act. In sum, policy preferences that were closely related to the highest-profile case decided in 2012 strongly correlate with warmth toward the Court and with one major reform measure.

**Fig 2:**
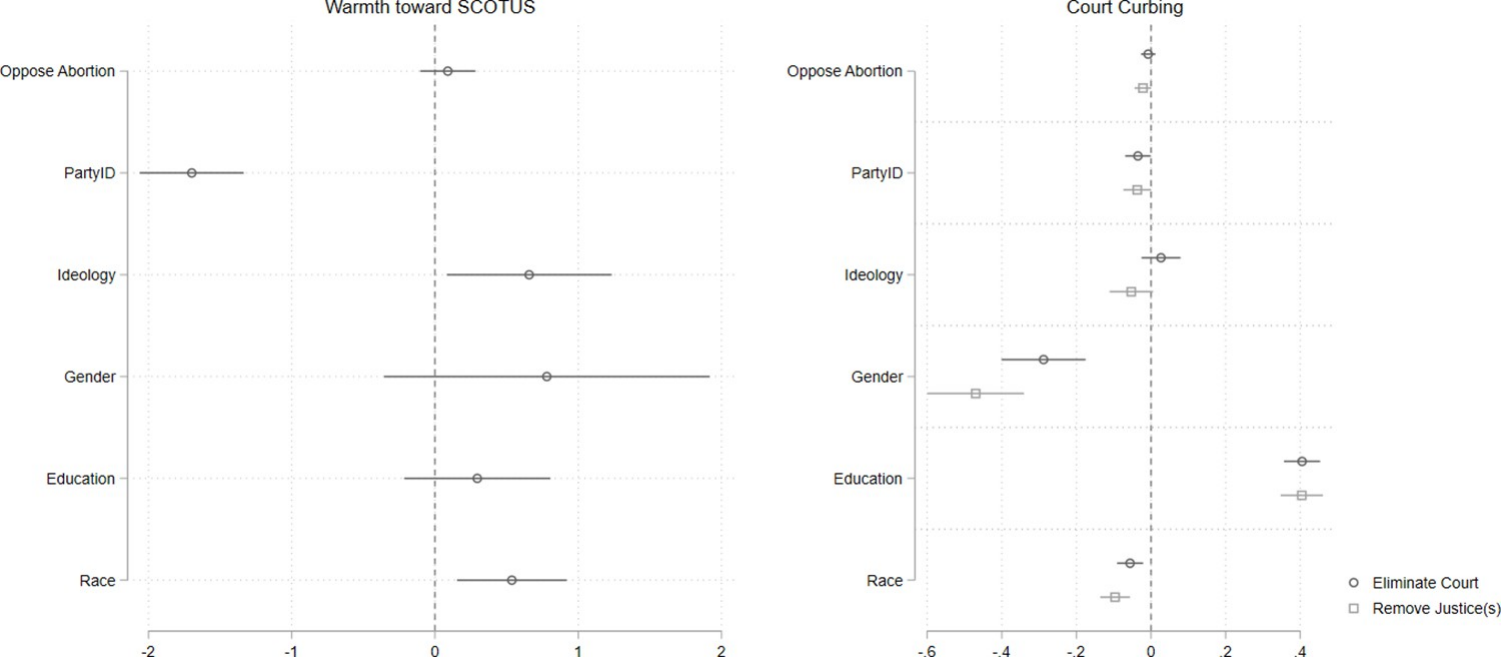
2012 abortion preferences do not predict attitudes toward SCOTUS in 2012. Point estimates with 95% confidence intervals from multivariate OLS models. Estimates in panel at left are based on *n =* 4,797 respondents. Estimates in panel at right are based on *n =* 4,756 (“eliminate court”) and *n =* 4,726 (“remove justice(s)).

Next, we conduct a placebo test using the same data. We fit the same models as those presented in Fig 1 but replace the variable indicating attitudes toward the Affordable Care Act with one that captures respondents’ abortion policy preferences. The logic of our argument implies that the public’s policy attitudes about issues in front of the Court drive their evaluation of decisions and broad views of the Court itself. Since there were no major decisions on abortion in 2012, if we are correct about the nature of attitudes toward the Court, we should find no strong relationship between abortion attitudes and assessments of the Court in 2012. Our analyses bear this expectation out. Fig 2 shows that abortion attitudes are unrelated to warmth toward the Court and to both Court reform measures. This finding increases confidence that case-specific policy attitudes crucially inform public assessments of the Supreme Court, and do so when these policy attitudes are salient.

### Study 2: Abortion and attitudes toward SCOTUS in 2022

In Study 2, we again assess the relationship between case-specific policy preferences and attitudes toward the Court using a different salient policy area a decade later–abortion policy. We use data from a UMass Amherst Poll, a 1,000-person nationally representative poll conducted by YouGov between May 5 and 9, 2022. In 2022, the highest profile decision was *Dobbs v*. *Jackson Women’s Health Organization* (2022, 597 US ___), in which the Court overturned *Roe v*. *Wade* (410 US 113) opening the door to unlimited regulation of abortion. The UMass survey was in the field after the *Dobbs* opinion was leaked to the public, but before it was officially announced by the Court. Our research design in Study 2 thus assumes that people are treating the leaked opinion essentially the same as they would an officially announced decision. We think this assumption is plausible because the messaging from the Court following the leak—which featured prominently in media coverage of *Dobbs*—insisted that the fact of the leak and the reaction to it would not affect the Court’s decision [34].

The UMass Poll did not include a question about respondents’ warmth toward SCOTUS but did ask about approval for the Supreme Court. Thus, we use approval of the Court as our dependent variable. The key independent variable hews very closely to the policy issue in *Dobbs*: whether the respondent thinks *Roe* should be overturned. We control for partisanship, ideology, race, gender, and education in an OLS regression model. Unadjusted models are presented in the supplement.

**Fig 3:**
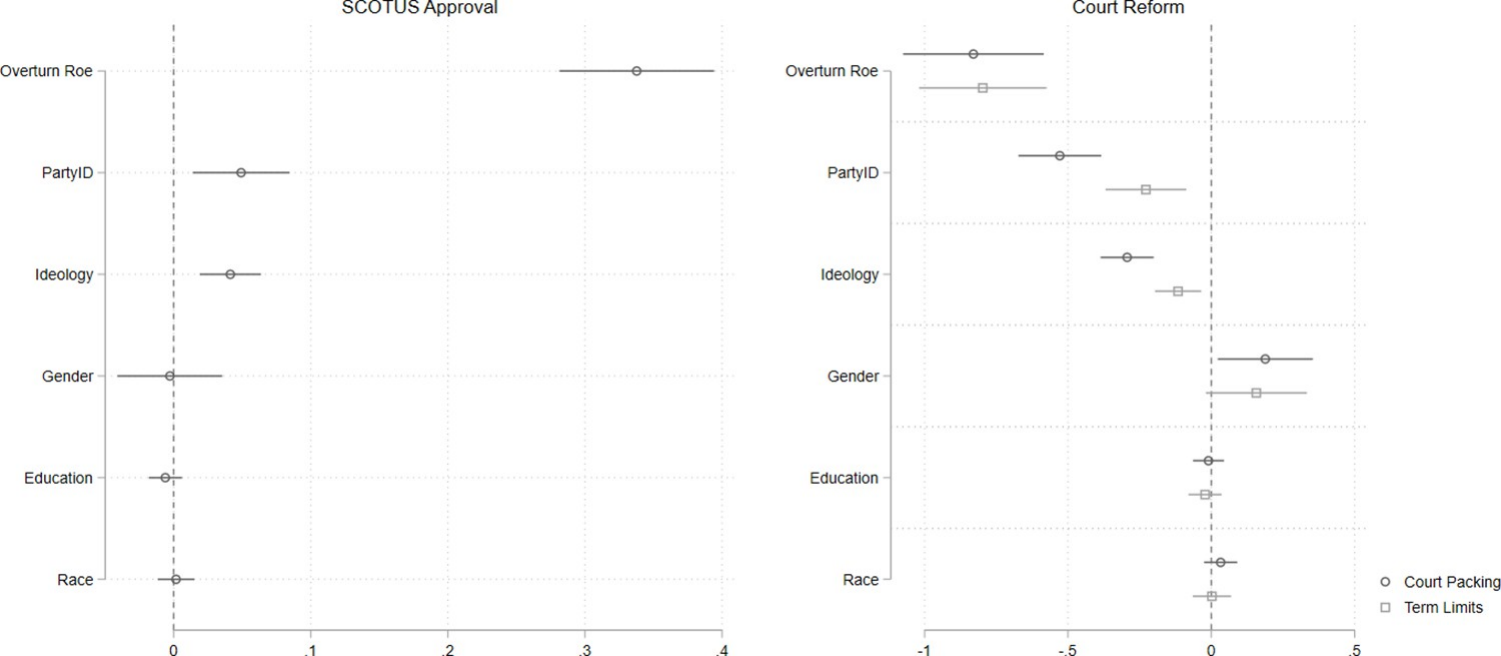
Policy preferences on salient issue before the court correlate strongly with approval of SCOTUS and attitudes about court reform. Point estimates with 95% confidence intervals from multivariate OLS models. Estimates in panel at left are based on *n =* 714 respondents; in panel at right, estimates are based on *n =* 768 respondents.

The findings are depicted in Fig 3; full models are presented in the supplement. Consistent with Study 1, we find that policy attitudes toward the issue captured in this very high-salience case strongly correlate with respondents’ approval of SCOTUS, net of controls. The coefficient on abortion policy attitudes is more than three-times the size of the coefficient on partisan identification. We also assess the relationship between policy attitudes and support for Court reforms—this time term limits and Court-packing, since respondents were asked about these specific reforms in the survey. The panel at right in Fig 3 shows that respondents who favor overturning *Roe* are much less supportive of both Court packing and term limits for justices than those who oppose overturning *Roe*.

**Fig 4:**
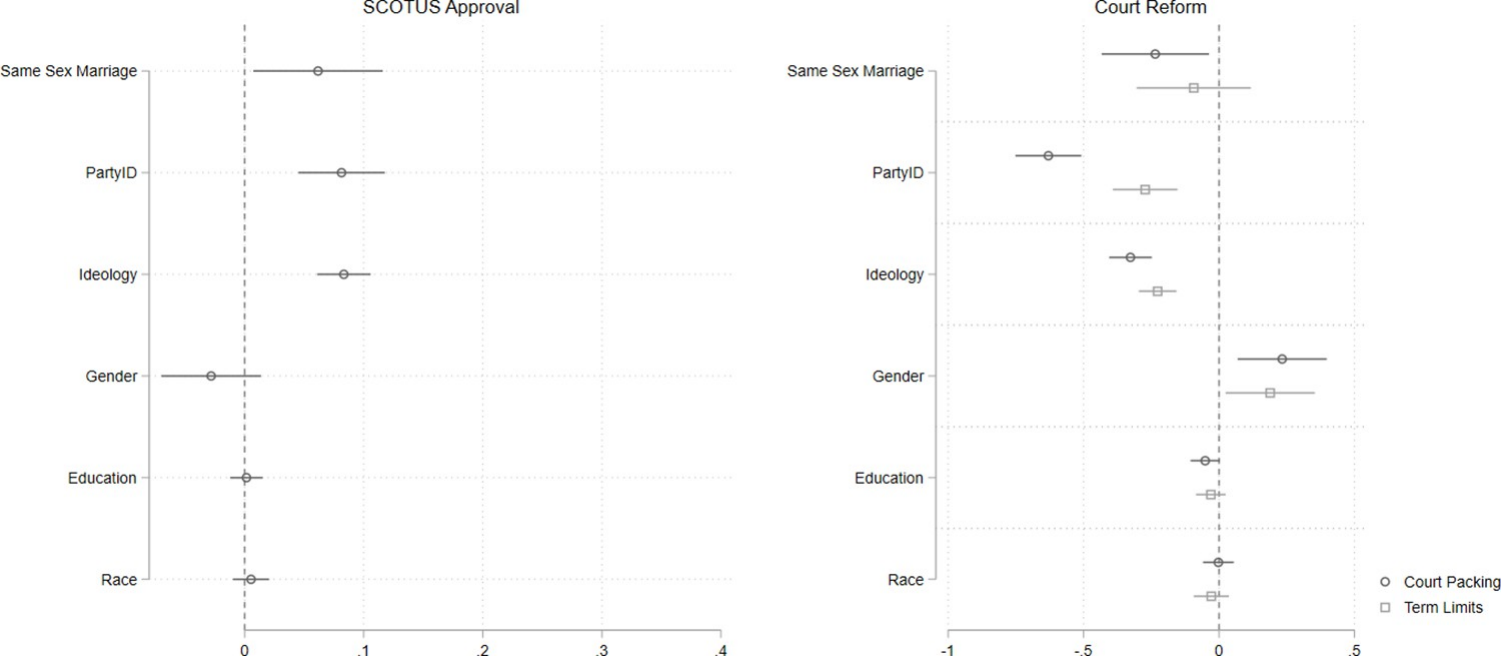
Policy preferences on issue not before the court do not correlate strongly with approval of SCOTUS or attitudes about court reform. Point estimates with 95% confidence intervals from multivariate OLS models. Estimates in panel at left are based on *n =* 795 respondents; in panel at left, estimates are based on *n =* 897 respondents.

To test the validity of this finding, we again conduct a placebo test, which is presented in Fig 4. The full models are presented in the supplement. In this placebo test, we replace the relevant policy attitude (abortion) with a policy question not relevant to the Court’s work in 2022: attitudes toward same-sex marriage. Here we see that favorability toward same-sex marriage does significantly correlate with SCOTUS approval (p = 0.026), but the coefficient is substantively small (about one-sixth the size of the *Roe* attitude in the true test, and about one-third the size of Republican identification). Similarly, we find only a weak relationship between attitudes toward same-sex marriage and support for Court-packing, and no relationship between the policy attitude and term limits. Here the pattern is not quite as clean as in Study 1, which is likely because media coverage of Roe and political elites expressly connected the overturning of *Roe* as a threat to same-sex marriage [35]. Taking the true test (Fig 3) and the placebo test (Fig 4) together, we find clear evidence that policy attitudes relevant to high-salience cases are strongly related to mass public assessments of the Court. As in Study 1, our findings here show that policy-specific attitudes are much more strongly correlated with respondents’ views about the Court than are their general partisan or ideological identifications.

To this point, we have presented evidence that when the Supreme Court decides high-profile cases, public policy preferences on the issue underlying the case in question strongly influence views toward the Court. Put simply, when people disagree with the policy outcome of Court decisions in high-profile cases, they feel more negatively toward the Court and express less approval compared to people who agree with the policy outcome. We also find evidence that people who disapprove are more supportive of Court curbing and Court reform measures compared to people who agree with the Court’s policy outputs.

In Study 3, we turn to experimental tests to confirm the causal relationships suggested by the observational studies, and to directly test the hypothesized mechanism of policy-based motivated reasoning.

### Study 3: Experimental confirmation of findings

Data for Study 3 comes from an original survey with an embedded experiment. The survey was fielded to a representative sample of 1,000 Americans by SSI/ResearchNow in May 2018. Treatment and question wordings, a randomization check, and the full analytic models are presented in the supplement. We gathered data on respondents’ pre-treatment policy preferences about two issues, the use of compulsory union dues for political contributions (low salience) and whether a baker should be able to refuse to provide services to a gay couple (high salience). The basic facts of these cases were modeled after *Janus v*. *AFSCME* (2018, 585 US ___) and *Masterpiece Cakeshop v*. *Colorado* (2018, 584 US ___), respectively. Then, we randomly assigned them to receive information about a Supreme Court decision touching on one of the policy areas they were asked about, or to a control condition where no information was provided. Crucially, because we think policy disagreement with the Court’s decision is going to drive attitudes toward the decision and the Court, and we randomly assigned respondents to agree or disagree with the Court’s decision. We did so by having two forms of the vignette for each case: one in which policy A won out, and another in which policy B won. This design was possible because the survey was in the field before the Court handed down its decisions in these real-world cases (*Masterpiece Cakeshop v*. *CCRC* was decided on June 4^th^, 2018, and *Janus v*. *AFSCME* was decided on June 27^th^, 2018.). Additionally, in both cases the outcome was very much in doubt, making either outcome plausible for participants.

The outcomes of interest here map onto the notion of institutional hybridity. To get at this we asked respondents two sets of questions after treatment. First, we asked them how “political” the decision they read about was, and how “legal” it was. Second, we asked how “political” and “legal” the Supreme Court itself is. The key independent variable is treatment to agree (or disagree) with the policy content of the Court’s decision.

**Fig 5:**
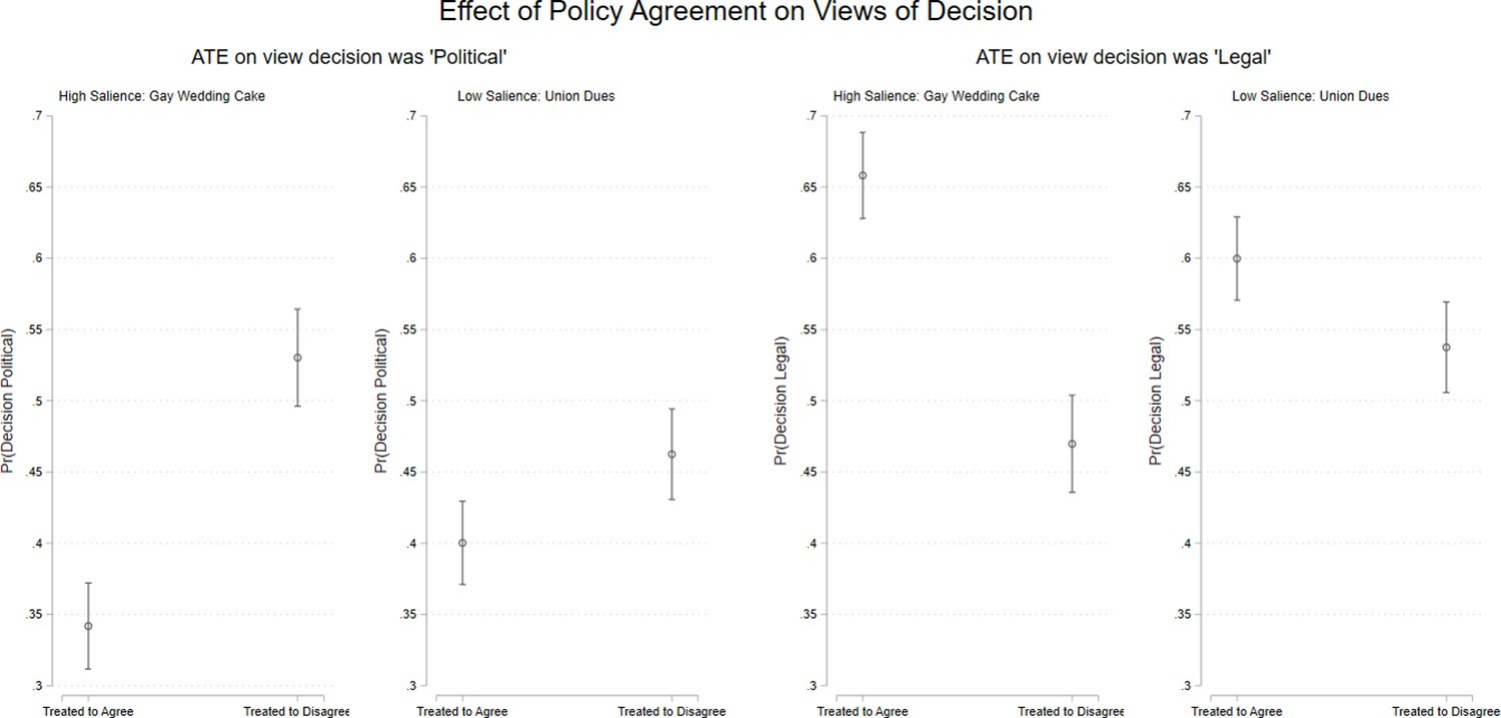
Decision disagreement causes changes in view of the decision. Point estimates (ATEs) with 95% confidence intervals. Predicted values from bivariate OLS models Estimates are based on *n* = 863 respondents.

The findings are depicted graphically in Fig 5. The analyses show large and highly significant effects of policy (dis)agreement on assessments of both the specific decision and views about the Supreme Court generally. The top panels show that respondents who were randomly assigned to disagree with the outcome in the case they read about reported viewing the decision as significantly *more “political” in nature* than those treated to agree. Conversely, those randomly assigned to disagree viewed the decision as *significantly less “legal” in nature*. Put simply, people chalk up decisions they disagree with to “politics,” but view decisions they like as being founded in the law. Additionally, we see that the size of the effect of disagreement is considerably different: the effect of policy disagreement on respondents’ assessment of how “political” or “legal” the decision was in the *Masterpiece CakeShop* decision is more than twice as large as the effect in the union dues case. This finding is suggestive that the size of the effect of policy agreement is conditioned by the political salience of the underlying issue.

**Fig 6:**
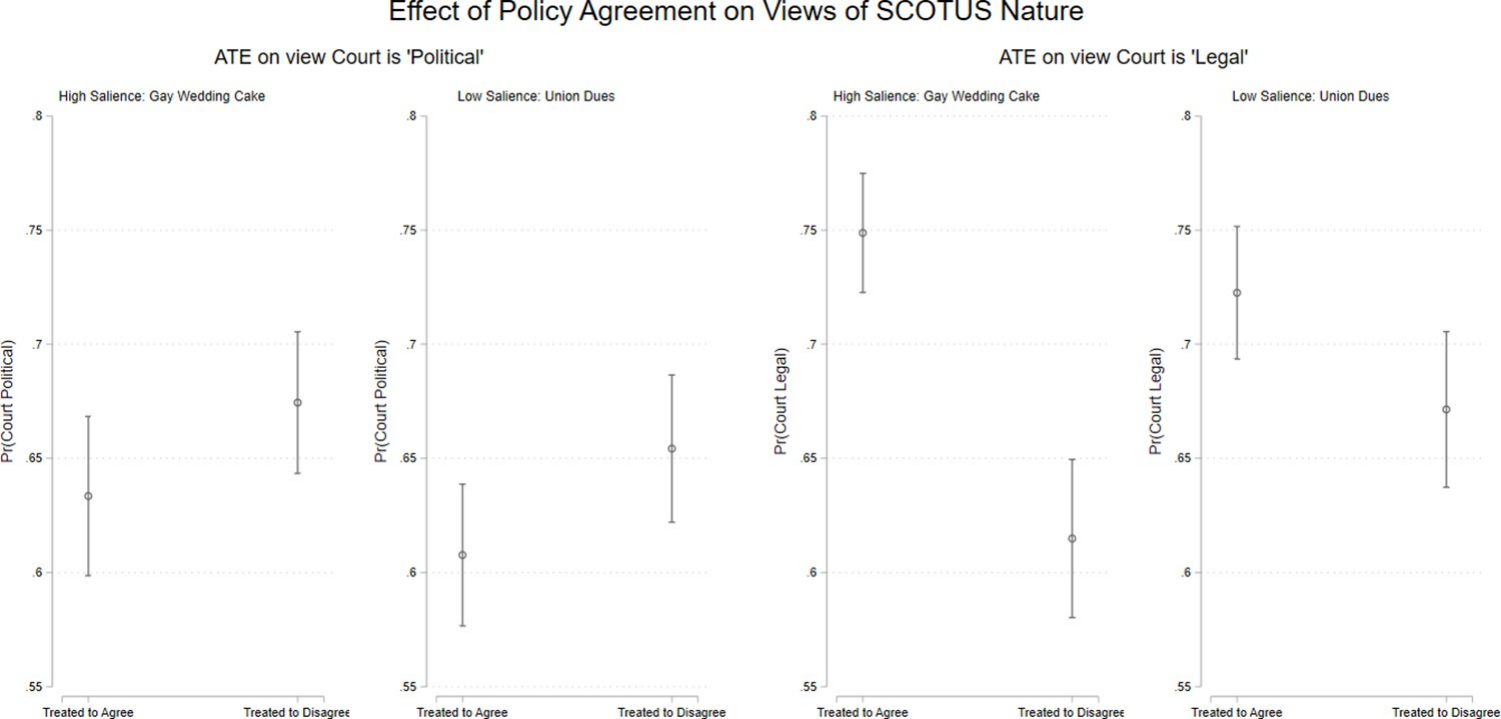
Decision disagreement causes changes in view of SCOTUS institutional nature. Point estimates (ATEs) with 95% confidence intervals. Predicted values from bivariate OLS models. Estimates are based on *n* = 863 respondents.

We expect that policy disagreement will also shape views of the Court itself. The plots in Fig 6 depict the effect of policy agreement on assessments of the Court’s institutional nature. Respondents who were randomly assigned to disagree with the Court’s decision reported viewing the Court itself as significantly more *“political”* and less *“legal”* in nature than those assigned to agree with the decision they read about. For the judgement about the Court being political the effect size was similar across the two cases, but for the “legal” judgment, the size of the effects were much larger for those reading about the higher salience issue.

Next, we turn to attitudes on Court curbing. Our survey did not include questions about Court packing or term limits, but it did include measures of attitudes on another prominent proposed reform: limiting the Court’s jurisdiction. The survey asked respondents whether we “ought to have stronger means of controlling” the Court, which captures respondents’ overall preferences regarding Court curbing.

**Fig 7:**
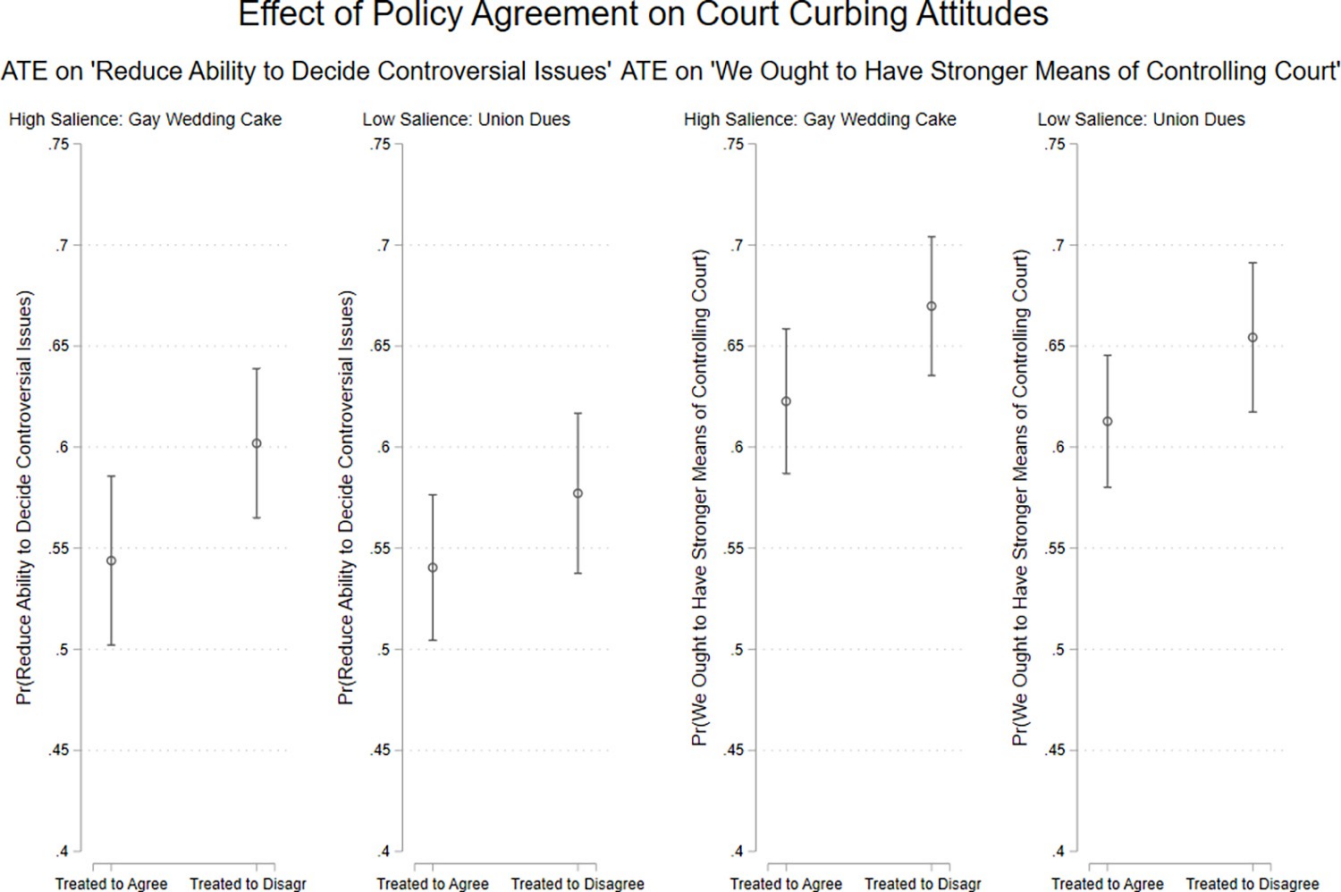
Decision disagreement increases support for court curbing. Point estimates (ATEs) with 95% confidence intervals. Predicted values from bivariate OLS models. Estimates are based on *n* = 863 respondents.

Consistent with the findings in the observational Studies 1 and 2, Fig 7 shows that those who were randomly assigned to see a decision they disagreed with were more likely to support reducing the Court’s jurisdiction (the predicted difference for the high salience case amounts to roughly 6 percentage points**)** and were more likely to say the Court should be subject to greater political control (by about 4 percentage points). Notably, though, the differences are only statistically significant for the higher salience issue.

In summary, Study 3 experimentally confirms that decision disagreement, especially in high-salience cases, causes people to view the Court and its work as more “political” in nature, and in turn support greater political oversight of the Court.

## Discussion

This paper makes several contributions. First, we draw on survey and experimental data covering more than a decade of public opinion and more than 6,000 respondents to show that policy agreement with Supreme Court outputs powerfully influences public views of the Court. Our findings generalize to the full population of American citizens over the age of 18, as all three samples are representative of adult U.S. citizens residing in the United States. Across multiple types of policy areas, we show that people who agree with Court policy outputs view the Court more warmly, express higher levels of job approval, and are less likely to support Court curbing or Court reform, compared to people who disagree with the Court’s policy decisions.

Second, and more centrally, we argue that policy-based motivated reasoning explains the documented relationship between assessments of the Court and policy preferences. The Court’s hybrid institutional nature enables a unique type of motivated reasoning wherein individuals attribute decisions with which they agree to the Court’s legal nature, processes, and motivations, while allowing them to ascribe decisions they do not like to politics. We document this link between the Court’s institutional nature and its decisions in multiple surveys and with an original survey with an embedded experiment.

Third, we show that policy agreement, and assessments of the Court’s political-legal nature, inform public support for Court reform. People who disagree with outputs, and people who view the Court as more political, are more likely to support Court curbing and other reforms, compared to those who agree with outputs and view the Court as more legal in nature.

This type of motivated reasoning has the potential to undermine institutional accountability as well. One outcome of this type of motivated reasoning is to weaken the connection between the Court’s actual performance and evaluations—what Little, Schnakenberg, and Turner [11] call “desensitization.” The public might not evaluate the competence of the Court’s decisions if they shift the bases of their evaluation to whether the decision is political or not when they do not like a case outcome.

This last point suggests an important implication for the literature on the role played by procedural fairness in informing citizens’ views of judicial institutions. Much prior work on this topic argues that perceptions about procedural fairness dampen the negative effects of disagreement with decisions. Our findings show that people’s perceptions of the process itself (how “political” versus “legal” it is) are influenced by their instrumental policy preferences. This finding implies one of two things: that procedural evaluations and policy preferences are endogenous, or that policy preferences dominate procedural considerations. Assessing the relationship between policy and process considerations is a particularly promising avenue for future research.

Our final contribution is methodological in nature. The literature on public support for the Supreme Court has long debated whether policy preferences matter. Scholars arguing that agreement with the Court is irrelevant have seldom actually measured policy preferences, however. Instead, these designs assume that self-identified conservatives (liberals) favor conservative (liberal) Court decisions. While this is on average likely true, because policy preferences only weakly correlate with political ideology and partisanship, this assumption causes us to misapprehend the preferences of many members of the public [14]. Failure to measure actual policy preferences, then, has likely led to errant conclusions about the nature of the relationship between policy preferences and support for the Court. Thus, in future work, it is crucial to measure policy preferences rather than assume them.

These contributions noted, we must also acknowledge the limitations of this study. First, while our studies demonstrate the effects of motivated reasoning, we do not experimentally manipulate individuals’ motivations (e.g., to be accurate or consistent). Second, although we vary salience in study 3, both of the cases used there are relatively low-attention for average members of the mass public. It is likely that our experimental results in study 3 underestimate the effects of motivated reasoning in very high-salience cases–issues which members of the public strongly care about. Third, our research design does not allow us to test the possibility that public preferences over legal interpretation or the meaning of law could matter independently of policy preferences. For example, it is theoretically possible that citizens’ views about the Affordable Care Act are endogenous to, or even downstream of, views about the nature of Congress’ authority to regulate commerce. Future work should test the possibility of independent effects of legal preferences. Finally, as noted above, our findings here are generalizable to the American public. This is both a strength and a limitation of our study. In general, Americans pay very little attention to the Supreme Court [31]. As a result, public views of the Court are typically mediated by elites such as politicians and members of the media. Future work, then, would do well to consider whether motivated reasoning drives the responses of these elites to Court decisions.

By shedding light on this unique form of motivated reasoning enabled by the Supreme Court’s hybrid institutional nature, we have directly linked citizens’ policy preferences to their institutional assessments of the Court. In doing so, our work suggests that the present Court’s penchant for handing down unpopular decisions will significantly undercut its standing among the public and fuel support for judicial reform.

## Methods

This research was categorized as Exempt by the Princeton University Institutional Review Board (protocol 10537) and the Syracuse University Institutional Review Board (protocol 17–081), and complied with all standards for research involving human subjects. Informed consent was obtained to participate in the study. The participants did not receive monetary compensation for their participation. The authors do not have access to information that could identify individual participants during or after data collection. In all cases, all valid survey responses were included in the analyses. All analyses were conducted using Stata (version 17). All data, materials, and code are available at https://doi.org/10.4231/0BTG-8D39.

### ANES survey data

The ANES asks respondents to rate political institutions, parties, and figures on a feeling thermometer ranging from 0 (very cold, unfavorable) to 100 (very warm, favorable). We used 2012 ANES data on the feeling thermometer ratings for the Supreme Court to capture support for the Court. Additionally, the 2012 ANES was especially useful for this study because it included two unusual questions capturing respondents’ views about eliminating the Supreme Court and replacing justices. These three questions serve as our dependent variables. ANES also asks a variety of questions about policy issues thought to be relevant to the contemporaneous election cycle. In 2012, ANES included questions about both the Affordable Care Act and abortion, which we used to construct the key independent variable in the test of our theory and the placebo test, respectively. Full question wordings are available in the supplement. The American National Election Study in 2012 interviewed 5,916 respondents using an address-based sample using a dual mode (face-to-face and Internet) study. The dual mode used separate samples of FTF and Internet respondents. All Internet respondents were members of KnowledgePanel, a panel of regular survey respondents maintained by survey firm GfK. All analyses include post-stratification weights provided by ANES to enable valid inferences about the population.

### UMass Poll survey data

To test our expectations in more recent data, we drew on the 2022 Umass Poll [36]. This iteration of the UMass Poll included questions on respondents’ attitudes toward the Supreme Court, including job approval, and support for Court reforms, which we use as dependent variables. The relevant policy attitudes to test our expectations are captured by questions about the Supreme Court’s then-recent overturning of *Roe v*. *Wade* (test), and about same-sex marriage (placebo). Full question wordings are available in the supplement. The UMass survey had 1000 respondents interviewed by YouGov who were drawn to be representative of respondents in the 2019 American Community Survey on gender, age, race, and education. All analyses include post-stratification weights provided by UMass Poll to enable valid inferences about the population.

### SSI/ResearchNow survey data

This is a non-probability sample consisting of 1,000 Americans drawn from SSI’s panel. The survey was fielded between May 9 and May 21, 2018. After providing consent to participate, respondents were asked questions about their policy preferences on the relevant issues and then were randomly assigned to read a vignette about one (of two) fictitious Supreme Court decisions. After treatment, respondents answered a series of questions about the decision described in the vignette, and about the Court itself. Survey responses were recorded using Qualtrics survey software. Full question wording is available in the supplement.

## Supporting information

S1 Table Table with full models supporting Fig 1. (DOCX)

S2 Table Table with unadjusted models demonstrating robustness of Fig 1. (DOCX)

S3 Table Table with full models supporting Fig 2. (DOCX)

S4 Table Table with unadjusted models supporting Fig 2. (DOCX)

S5 Table Table with full models supporting Fig 3. (DOCX)

S6 Table Table with unadjusted models demonstrating robustness of Fig 3. (DOCX)

S7 Table Table with full models supporting Fig 4. (DOCX)

S8 Table Table with unadjusted models supporting Fig 4. (DOCX)

S9 Table Table with randomization check for Study 3 (SSI data). (DOCX)

S10 Table Table with full models supporting Fig 5. (DOCX)

S11 Table Table with full models supporting Fig 6. (DOCX)

S12 Table Table with full models supporting Fig 7. (DOCX)

S1 Appendix Appendix for Study 1. (DOCX)

S2 Appendix Appendix for Study 2. (DOCX)

S3 Appendix Appendix for Study 3. (DOCX)
