# Supplementary material for: Institutional hybridity and policy-motivated reasoning structure public evaluations of the Supreme Court
Source: PLoS One. 2023 Nov 22;18(11):e0294525. doi: 10.1371/journal.pone.0294525 (PMC10664892; doi:10.1371/journal.pone.0294525)
Supplement: S1 Table — (DOCX) [file pone.0294525.s001.docx]

**S1. Table with Full models supporting Figure 1**

|  | Warmth toward | Eliminate | Remove |
| --- | --- | --- | --- |
| VARIABLES | SCOTUS | SCOTUS | SCOTUS Justice |
| Favor ACA | 9.44*** | 0.15 | 0.46*** |
|  | (1.01) | (0.10) | (0.12) |
| Party ID | -0.88*** | -0.02 | -0.00 |
|  | (0.20) | (0.02) | (0.02) |
| Ideology | 1.28*** | 0.02 | -0.04 |
|  | (0.28) | (0.03) | (0.03) |
| Gender | 0.55 | -0.29*** | -0.47*** |
|  | (0.58) | (0.06) | (0.07) |
| Education | 0.16 | 0.40*** | 0.40*** |
|  | (0.26) | (0.02) | (0.03) |
| Race | 0.48* | -0.06** | -0.10*** |
|  | (0.19) | (0.02) | (0.02) |
| Constant | 47.87*** | 4.22*** | 3.20*** |
|  | (1.96) | (0.19) | (0.22) |
| Observations | 4,802 | 4,757 | 4,722 |
| R-squared | 0.05 | 0.06 | 0.07 |

Standard errors in parentheses, *** p<0.001, ** p<0.01, * p<0.05
