# Supplementary material for: Institutional hybridity and policy-motivated reasoning structure public evaluations of the Supreme Court
Source: PLoS One. 2023 Nov 22;18(11):e0294525. doi: 10.1371/journal.pone.0294525 (PMC10664892; doi:10.1371/journal.pone.0294525)
Supplement: S2 Table — (DOCX) [file pone.0294525.s002.docx]

**S2. Table with Unadjusted models demonstrating robustness of Figure 1**

|  | Warmth toward | Eliminate | Remove |
| --- | --- | --- | --- |
| VARIABLES | SCOTUS | SCOTUS | SCOTUS Justice |
| Favor ACA | 10.53*** | 0.07 | 0.39*** |
|  | (0.72) | (0.08) | (0.09) |
| Constant | 51.91*** | 4.93*** | 3.31*** |
|  | (0.49) | (0.05) | (0.06) |
| Observations | 5,375 | 5,311 | 5,292 |
| R-squared | 0.04 | 0.00 | 0.00 |

Standard errors in parentheses, *** p<0.001, ** p<0.01, * p<0.05
