# Supplementary material for: Institutional hybridity and policy-motivated reasoning structure public evaluations of the Supreme Court
Source: PLoS One. 2023 Nov 22;18(11):e0294525. doi: 10.1371/journal.pone.0294525 (PMC10664892; doi:10.1371/journal.pone.0294525)
Supplement: S3 Table — (DOCX) [file pone.0294525.s003.docx]

**S3. Table with Full models supporting Figure 2**

|  | Warmth toward | Eliminate | Remove |
| --- | --- | --- | --- |
| VARIABLES | SCOTUS | SCOTUS | SCOTUS Justice |
| Oppose Abortion | 0.09 | -0.01 | -0.02 |
|  | (0.10) | (0.01) | (0.01) |
| Party ID | -1.70*** | -0.04* | -0.04 |
|  | (0.19) | (0.02) | (0.02) |
| Ideology | 0.66* | 0.03 | -0.05 |
|  | (0.29) | (0.03) | (0.03) |
| Gender | 0.78 | -0.29*** | -0.47*** |
|  | (0.58) | (0.06) | (0.07) |
| Education | 0.30 | 0.40*** | 0.40*** |
|  | (0.26) | (0.02) | (0.03) |
| Race | 0.54** | -0.06** | -0.10*** |
|  | (0.19) | (0.02) | (0.02) |
| Constant | 57.07*** | 4.37*** | 3.71*** |
|  | (1.70) | (0.17) | (0.19) |
| Observations | 4,797 | 4,756 | 4,726 |
| R-squared | 0.03 | 0.06 | 0.07 |

Standard errors in parentheses, *** p<0.001, ** p<0.01, * p<0.05
