# Supplementary material for: Institutional hybridity and policy-motivated reasoning structure public evaluations of the Supreme Court
Source: PLoS One. 2023 Nov 22;18(11):e0294525. doi: 10.1371/journal.pone.0294525 (PMC10664892; doi:10.1371/journal.pone.0294525)
Supplement: S4 Table — (DOCX) [file pone.0294525.s004.docx]

**S4. Table with Unadjusted models supporting Figure 2**

|  | Warmth toward | Eliminate | Remove |
| --- | --- | --- | --- |
| VARIABLES | SCOTUS | SCOTUS | SCOTUS Justice |
| Oppose Abortion | -0.15 | -0.03** | -0.05*** |
|  | (0.08) | (0.01) | (0.01) |
| Constant | 58.33*** | 5.10*** | 3.78*** |
|  | (0.48) | (0.05) | (0.06) |
| Observations | 5,371 | 5,310 | 5,296 |
| R-squared | 0.00 | 0.00 | 0.01 |

Standard errors in parentheses, *** p<0.001, ** p<0.01, * p<0.05
