# Supplementary material for: Institutional hybridity and policy-motivated reasoning structure public evaluations of the Supreme Court
Source: PLoS One. 2023 Nov 22;18(11):e0294525. doi: 10.1371/journal.pone.0294525 (PMC10664892; doi:10.1371/journal.pone.0294525)
Supplement: S5 Table — (DOCX) [file pone.0294525.s005.docx]

**S5. Table with Full models supporting Figure 3**

|  |  |  |  |
| --- | --- | --- | --- |
| VARIABLES | SCOTUS Approval | Court Packing | Term Limits |
| Overturn Roe | 0.34*** | -0.83*** | -0.80*** |
|  | (0.03) | (0.12) | (0.11) |
| Party ID | 0.05** | -0.53*** | -0.23** |
|  | (0.02) | (0.07) | (0.07) |
| Ideology | 0.04*** | -0.29*** | -0.12** |
|  | (0.01) | (0.05) | (0.04) |
| Gender | -0.00 | 0.19* | 0.16 |
|  | (0.02) | (0.08) | (0.09) |
| Education | -0.01 | -0.01 | -0.02 |
|  | (0.01) | (0.03) | (0.03) |
| Race | 0.00 | 0.03 | 0.00 |
|  | (0.01) | (0.03) | (0.03) |
| Constant | 0.09 | 4.24*** | 4.45*** |
|  | (0.05) | (0.21) | (0.22) |
| Observations | 714 | 768 | 768 |
| R-squared | 0.45 | 0.48 | 0.24 |

Standard errors in parentheses, *** p<0.001, ** p<0.01, * p<0.05
