# Supplementary material for: Institutional hybridity and policy-motivated reasoning structure public evaluations of the Supreme Court
Source: PLoS One. 2023 Nov 22;18(11):e0294525. doi: 10.1371/journal.pone.0294525 (PMC10664892; doi:10.1371/journal.pone.0294525)
Supplement: S6 Table — (DOCX) [file pone.0294525.s006.docx]

**S6. Table with Unadjusted models demonstrating robustness of Figure 3**

|  |  |  |  |
| --- | --- | --- | --- |
| VARIABLES | SCOTUS Approval | Court Packing | Term Limits |
| Overturn Roe | 0.46*** | -1.84*** | -1.23*** |
|  | (0.02) | (0.09) | (0.09) |
| Constant | 0.18*** | 3.59*** | 4.22*** |
|  | (0.01) | (0.06) | (0.05) |
| Observations | 775 | 836 | 836 |
| R-squared | 0.42 | 0.34 | 0.20 |

Standard errors in parentheses, *** p<0.001, ** p<0.01, * p<0.05
