# Supplementary material for: Institutional hybridity and policy-motivated reasoning structure public evaluations of the Supreme Court
Source: PLoS One. 2023 Nov 22;18(11):e0294525. doi: 10.1371/journal.pone.0294525 (PMC10664892; doi:10.1371/journal.pone.0294525)
Supplement: S7 Table — (DOCX) [file pone.0294525.s007.docx]

**S7. Table with Full Models supporting Figure 4**

|  |  |  |  |
| --- | --- | --- | --- |
| VARIABLES | SCOTUS Approval | Court Packing | Term Limits |
| Same Sex Marriage | 0.06* | -0.24* | -0.09 |
|  | (0.03) | (0.10) | (0.11) |
| Party ID | 0.08*** | -0.63*** | -0.27*** |
|  | (0.02) | (0.06) | (0.06) |
| Ideology | 0.08*** | -0.33*** | -0.23*** |
|  | (0.01) | (0.04) | (0.04) |
| Gender | -0.03 | 0.23** | 0.19* |
|  | (0.02) | (0.08) | (0.08) |
| Education | 0.00 | -0.05 | -0.03 |
|  | (0.01) | (0.03) | (0.03) |
| Race | 0.01 | -0.00 | -0.03 |
|  | (0.01) | (0.03) | (0.03) |
| Constant | -0.02 | 4.61*** | 4.67*** |
|  | (0.06) | (0.23) | (0.24) |
| Observations | 795 | 897 | 897 |
| R-squared | 0.27 | 0.38 | 0.15 |

Standard errors in parentheses, *** p<0.001, ** p<0.01, * p<0.05
