# Supplementary material for: Institutional hybridity and policy-motivated reasoning structure public evaluations of the Supreme Court
Source: PLoS One. 2023 Nov 22;18(11):e0294525. doi: 10.1371/journal.pone.0294525 (PMC10664892; doi:10.1371/journal.pone.0294525)
Supplement: S8 Table — (DOCX) [file pone.0294525.s008.docx]

**S8. Table with Unadjusted models supporting Figure 4**

|  |  |  |  |
| --- | --- | --- | --- |
| VARIABLES | SCOTUS Approval | Court Packing | Term Limits |
| Same Sex Marriage | 0.25*** | -1.13*** | -0.61*** |
|  | (0.02) | (0.09) | (0.09) |
| Constant | 0.03 | 4.39*** | 4.51*** |
|  | (0.03) | (0.13) | (0.12) |
| Observations | 870 | 997 | 997 |
| R-squared | 0.12 | 0.13 | 0.05 |

Standard errors in parentheses, *** p<0.001, ** p<0.01, * p<0.05
