# Supplementary material for: Institutional hybridity and policy-motivated reasoning structure public evaluations of the Supreme Court
Source: PLoS One. 2023 Nov 22;18(11):e0294525. doi: 10.1371/journal.pone.0294525 (PMC10664892; doi:10.1371/journal.pone.0294525)
Supplement: S9 Table — (DOCX) [file pone.0294525.s009.docx]

**S9. Table with Randomization Check for Study 3 (SSI data)**

|  | Treatment |
| --- | --- |
| VARIABLES | Condition |
| Male | 0.07 |
|  | (0.06) |
| Age | -0.00 |
|  | (0.00) |
| Education Level | -0.01 |
|  | (0.02) |
| Republican | -0.00 |
|  | (0.08) |
| Conservative | 0.03 |
|  | (0.02) |
| White | -0.04 |
|  | (0.06) |
| Constant | 0.96*** |
|  | (0.14) |
| Observations | 934 |
| R-squared | 0.01 |

Standard errors in parentheses, *** p<0.001, ** p<0.01, * p<0.05
