# Supplementary material for: Institutional hybridity and policy-motivated reasoning structure public evaluations of the Supreme Court
Source: PLoS One. 2023 Nov 22;18(11):e0294525. doi: 10.1371/journal.pone.0294525 (PMC10664892; doi:10.1371/journal.pone.0294525)
Supplement: S10 Table — (DOCX) [file pone.0294525.s010.docx]

**S10. Table with Full models supporting Figure 5**

|  | *Masterpiece* | *Janus* | *Masterpiece* | *Janus* |
| --- | --- | --- | --- | --- |
| VARIABLES | Decision Political | Decision Political | Decision Legal | Decision Legal |
| Treated to Disagree | 0.19*** | 0.05* | -0.19*** | -0.06** |
|  | (0.02) | (0.02) | (0.02) | (0.02) |
| Constant | 0.34*** | 0.61*** | 0.66*** | 0.60*** |
|  | (0.02) | (0.02) | (0.02) | (0.01) |
| Observations | 418 | 445 | 418 | 445 |
| R-squared | 0.14 | 0.01 | 0.14 | 0.02 |

Robust standard errors in parentheses, *** p<0.001, ** p<0.01, * p<0.05, ^+^ p<0.1
