# Supplementary material for: Institutional hybridity and policy-motivated reasoning structure public evaluations of the Supreme Court
Source: PLoS One. 2023 Nov 22;18(11):e0294525. doi: 10.1371/journal.pone.0294525 (PMC10664892; doi:10.1371/journal.pone.0294525)
Supplement: S12 Table — (DOCX) [file pone.0294525.s012.docx]

**S12. Table with Full models supporting Figure 7**

|  | *Masterpiece* | *Janus* | *Masterpiece* | *Janus* |
| --- | --- | --- | --- | --- |
| VARIABLES | Jurisdiction Stripping | Jurisdiction Stripping | Increase Control | Increase Control |
| Treated to Disagree | 0.06* | 0.04 | 0.05^+^ | 0.04^+^ |
|  | (0.03) | (0.03) | (0.03) | (0.03) |
| Constant | 0.54*** | 0.54*** | 0.62*** | 0.61*** |
|  | (0.02) | (0.02) | (0.02) | (0.02) |
| Observations | 418 | 445 | 418 | 445 |
| R-squared | 0.01 | 0.00 | 0.01 | 0.01 |

Robust standard errors in parentheses, *** p<0.001, ** p<0.01, * p<0.05, ^+^ p<0.1
