# Supplementary material for: Institutional hybridity and policy-motivated reasoning structure public evaluations of the Supreme Court
Source: PLoS One. 2023 Nov 22;18(11):e0294525. doi: 10.1371/journal.pone.0294525 (PMC10664892; doi:10.1371/journal.pone.0294525)
Supplement: S1 Appendix — (DOCX) [file pone.0294525.s013.docx]

**S1. Appendix for Study 1**

**2012 ANES**

***Question Wording for Key Variables***

**Warmth toward Supreme Court** (DV 1)
We’d also like to get your feelings about some groups in American society. When I read the name of a group, we’d like you to rate it with what we call a feeling thermometer. Ratings between 50 degrees-100 degrees mean that you feel favorably and warm toward the group; ratings between 0 and 50 degrees mean that you don’t feel favorably towards the group and that you don’t care too much for that group. If you don’t feel particularly warm or cold toward a group you would rate them at 50 degrees. If we come to a group you don’t know much about, just tell me and we’ll move on to the next one.
([And] still using the thermometer, how would you rate [the following]:) The Supreme Court

Response scale: 0 – 100 Degrees

**Note**: Group thermometers were administered in random order.

**Eliminate SCOTUS** (DV 2)

If the U.S. Supreme Court started making a lot of decisions that most people disagree with, would you favor, oppose, or neither favor nor oppose doing away with the Supreme Court altogether?

1. Favor
2. Oppose
3. Neither favor or oppose

Do you [favor/oppose] that strongly or not strongly?

1. Strongly
2. Not strongly

**Remove SCOTUS Justices** (DV 3)

Do you favor, oppose, or neither favor nor oppose removing judges from the U.S. Supreme Court if those judges consistently make decisions that a majority of Americans oppose?

1. Favor
2. Oppose
3. Neither favor nor oppose

Do you [favor/oppose] that strongly or not strongly?

1. Strongly
2. Not strongly

**Attitudes toward Affordable Care Act** (key IV)

Do you favor, oppose, or neither favor nor oppose the health care reform law passed in 2010? This law requires all Americans to buy health insurance and requires health insurance companies to accept everyone.

1. Favor
2. Oppose
3. Neither favor nor oppose

Do you [favor/oppose] that [a great deal, moderately, or a little / a little, moderately, or a great deal]?

1. A great deal
2. Moderately
3. A little

**Attitudes toward Abortion** (placebo IV)

Next, do you favor, oppose, or neither favor nor oppose abortion being legal if the woman chooses to have one?

1. Favor
2. Oppose
3. Neither favor nor oppose

Do you [favor/oppose] that [a great deal, moderately, or a little / a little, moderately, or a great deal]?

1. A great deal
2. Moderately
3. A little
