# Supplementary material for: Institutional hybridity and policy-motivated reasoning structure public evaluations of the Supreme Court
Source: PLoS One. 2023 Nov 22;18(11):e0294525. doi: 10.1371/journal.pone.0294525 (PMC10664892; doi:10.1371/journal.pone.0294525)
Supplement: S2 Appendix — (DOCX) [file pone.0294525.s014.docx]

**S2. Appendix for Study 2**

**2022 UMASS Poll**

***Question Wording for Key Variables***

**SCOTUS Job Approval** (DV 1)

Do you approve or disapprove of the way each is doing their job? … U.S. Supreme Court

1. Strongly approve
2. Somewhat approve
3. Somewhat disapprove
4. Strongly disapprove
5. Not sure

**Changes to the Court (Court Packing & Term Limits –** DVs 2 & 3**)**

Please indicate your level of support for the following proposals.

Increasing the number of justices on the U.S. Supreme Court

1. Strongly support
2. Support
3. Neither support nor oppose
4. Oppose
5. Strongly oppose

Establishing term limits for justices who serve on the U.S. Supreme Court.

1. Strongly support
2. Support
3. Neither support nor oppose
4. Oppose
5. Strongly oppose

**Approve overturning *Roe*** (key IV)

Recent reporting indicates that the U.S. Supreme Court is about to overturn *Roe v. Wade*, the landmark decision that established a woman’s right to choose to have an abortion. This decision would allow individual states to pass laws banning abortion. Do you think that the Supreme Court should overturn Roe v. Wade?

1. Yes, the Supreme Court should overturn *Roe v. Wade*
2. No, the Supreme Court should not overturn *Roe v. Wade*
3. Don’t know

**Same Sex Marriage** (placebo IV)

Do you favor or oppose allowing gays and lesbians to marry legally?

1. Favor
2. Oppose
